# The effect of omega-3 and vitamin D co-supplementation on glycemic control and lipid profiles in reproductive-aged women with pre-diabetes and hypovitaminosis D: a randomized controlled trial

**DOI:** 10.1186/s13098-020-00549-9

**Published:** 2020-05-12

**Authors:** Masoumeh Rajabi-Naeeni, Mahrokh Dolatian, Mostafa Qorbani, Amir Abbas Vaezi

**Affiliations:** 1 Department of Midwifery and Reproductive Health, School of Nursing and Midwifery, Student Research Committee, Shahid Beheshti University of Medical Sciences, Tehran, Iran; 2 Midwifery and Reproductive Health Research Center, Department of Midwifery and Reproductive Health, School of Nursing and Midwifery, Shahid Beheshti University of Medical Sciences, Tehran, Iran; 3 Non-communicable Diseases Research Center, Alborz University of Medical Sciences, Karaj, Iran; 4 Chronic Diseases Research Center, Endocrinology and Metabolism Population Sciences Institute, Tehran University of Medical Sciences, Tehran, Iran; 5 Department of Internal Medicine, School of Medicine, Alborz University of Medical Sciences, Karaj, Iran

**Keywords:** Vitamin D, Omega-3 fatty acids, LDL-C, HDL-C, Insulin resistance, Pre-diabetic state, Type 2 diabetes mellitus

## Abstract

**Background:**

Prediabetes can predispose the individual to type 2 diabetes in the long-term. The present study was conducted to determine the effectiveness of vitamin D and omega-3 co-supplementation on glycemic control and serum lipid profiles in women of reproductive age with prediabetes and hypovitaminosis D.

**Methods:**

The present factorial, triple-blind, clinical trial was conducted on 168 women of reproductive age with prediabetes and hypovitaminosis D. The participants were assigned to four groups based on block randomization method: the placebo group received omega-3 and vitamin D placebos; the omega-3 group took omega-3 supplements and vitamin D placebos; the vitamin D group received omega-3 placebos and vitamin D supplements and the co-supplementation group. The groups received every 2 weeks 50,000 IU pearls of vitamin D and twice-daily doses of 1000-mg omega-3 tablets or placebos for 8 weeks. Dietary intake, physical activity, anthropometric indices and blood biochemical tests were measured at the beginning and end of the study. Analysis was performed using two-way mixed ANOVA.

**Results:**

A significant reduction was observed in fasting glucose, insulin, homeostasis model assessment-beta cell function, weight and waist circumference in the co-supplementation group compared to the other three groups (P < 0.05). Moreover, high-density lipoprotein-cholesterol levels increased significantly in the co-supplementation group compared to the other three groups (P < 0.05). Despite the fact that homeostasis model assessment-insulin resistance, total cholesterol, triglyceride and low-density lipoprotein-cholesterol levels decreased after intervention in the co-supplementation group, there was no significant difference between the groups in these outcomes.

**Conclusion:**

Vitamin D and omega-3 co-supplementation improved fasting serum glucose, insulin, high-density lipoprotein-cholesterol level, homeostasis model assessment-beta cell function, weight and waist circumference in women of reproductive age with prediabetes and hypovitaminosis D. This co-supplementation can therefore be recommended for glycemic control in these women.

*Trial registration* Iranian Registry of Clinical Trials Code: IRCT20100130003226N17. Registered on 9 Feb. 2019

## Background

Prediabetes is a transient stage between normal blood glucose level and diabetes type 2. Prediabetic people are at risk of progressing toward diabetes [1]. Every year 5 to 10 percent of this group develops diabetes type 2 [2]. A total of 318 million people were reported to have prediabetes in 2015 around the world, which is anticipated to increase to 471 million by 2035 [3]. Studies have reported the incidence of nephropathy, retinopathy and macro vascular problems in prediabetes stage [2]. Insulin resistance and hypovitaminosis D adversely affects women’s reproductive health and causes menstrual changes, impaired fertility, sexual dysfunction, urinary and vaginal infections, urinary incontinence and depression. An increased risk of breast and ovarian cancer has also been reported with increased insulin resistance [4–6]. During pregnancy this group is at risk of gestational diabetes [7].

Studies have confirmed the beneficial effects of weight loss, increased physical activity, and use of medications such as metformin in increasing insulin sensitivity [8–10]. However, inconsistent adherence to the medication regimen due to the side effects experienced and the difficulties in maintaining weight and physical activity in the long term further reveal the need for an effective and safe intervention to control this problem [10, 11].

The use of supplements has become very popular in recent years. There has been some evidence suggesting that vitamin D deficiency has a role in the development of diabetes. The receptors of this vitamin are present in the organs involved glucose-metabolism, such as the pancreatic beta cells, adipose tissue and liver [12, 13] and can therefore increase insulin secretion and stimulate glucose transfer [14, 15]. Nonetheless, various trial studies have reported different effects of vitamin D in pre-diabetic individuals [16].

Moreover, subclinical inflammation is regarded as one of the pathological mechanisms of developing diabetes type 2. As a bioactive anti-inflammatory agent, omega-3 appears to be effective on a large number of physiological mechanisms. Therefore, using omega-3 can be considered as a strategy for delaying progress to diabetes type 2 in pre-diabetic individuals [17–19]. Studies have yielded contradictory results on the effects of omega-3 on insulin sensitivity [20–23]. Some studies have revealed positive effects for vitamin D and omega-3 co-supplementation on the metabolic status of people with gestational diabetes and diabetes type I [24, 25]. Given these conflicting reports and also since the effect of the concurrent intake of these two supplements has not been assessed in pre-diabetic subjects, the present study was conducted to determine the effectiveness of vitamin D and omega-3 co-supplementation on glucose control and the serum lipid profile in women of reproductive age with prediabetes and hypovitaminosis D.

## Methods

### Study design and participants

In this factorial, triple-blind, clinical trial, 168 pre-diabetic women aged 15–50 years with hypovitaminosis D using stratified permuted block randomization method were assigned to four study groups for 8 weeks. The study was approved by the Ethics Committee of Shahid Beheshti University of Medical Sciences and registered at the Iranian Registry of Clinical Trials under the code IRCT20100130003226N17. The study was conducted at Shahid Rastravesh Health Center Laboratory of Alborz University of Medical Sciences, Karaj, Iran, from March to August 2019. A detailed description of the study protocol has been published previously and here we briefly explain it [26].

The study inclusion criteria were: (1) Being a woman of reproductive age with prediabetes and a fasting glucose of 100–125 mg/dL [27]; (2) Serum vitamin D < 32 ng/mL [28]; (3) BMI < 30 kg/m^2^; and (4) Willingness to take part in the study. The exclusion criteria were: (1) Diabetes type 1 or 2 or other metabolic and underlying diseases; (2) Using herbal or chemical medications affecting serum glucose and lipids; (3) Having used vitamin D and omega-3 supplements over the last 6 months; (4) Using medications interacting with omega-3 and vitamin D; (5) Pregnancy or breastfeeding; and (6) Non-adherence to the medication administration protocols.

Sampling was carried out in two stages. All women of reproductive age, referred by the health centers of Karaj city to Shahid Rastravesh Health Center Laboratory for diabetes screening. In the first stage, women who had a fasting glucose of 100 to 125 mg/dl [27–29] were contacted over the phone. After a brief introduction, the researcher explained the study objectives to the women and those willing to take part and met the study criteria were invited to come to the laboratory. The second stage began after obtaining the women’s written consent. The participants then underwent fasting blood glucose and insulin, serum vitamin D and serum lipids tests, and those with confirmed test results showing the fasting glucose to be 100 to 125 mg/dl and serum vitamin D < 32 ng/mL entered the study [27, 28]. They were visited three times during this study. In the first visit, a blood test was taken from them and their anthropometric indices, including height, weight and waist circumference were measured. Then they completed a general questionnaire and the International Physical Activity Questionnaire—short form (IPAQ-SF). Afterwards, they received the blank three-day food record and a nutritionist briefed them how to complete the forms. Three days later, in the second visit, they submitted their three-day food record and if prediabetes and hypovitaminosis D confirmed by the test results, they were randomly assigned to any of the following four groups: The placebo group, the vitamin D group, the omega-3 group and finally the vitamin D and omega-3 co-supplementation group. At the end of the eighth week, blood tests, anthropometric measurements and IPAQ and three-day food records were repeated for the participants. The results pertaining to each participant were explained to the subjects in private at the end of the study.

### Randomization and Intervention

The participants were assigned to four groups by stratified random block sampling. Block randomization was done in equal block sizes of 4 to ensure the balance between groups. Stratified randomization was performed based on serum 25(OH)D concentration: Under 20 ng/mL and between 20 and 32 ng/mL [28–30]. An individual who is not involved in trial data collection and analysis carried out randomization. The intervention allocation was blinded to the study investigators, participants and statistical analyzer. The dosage of supplements was determined according to previous studies as follows: 50,000 IU of vitamin D (cholecalciferol) every 2 weeks and 1 g of omega-3 twice per day [24].

The vitamin D (IRC: 1228055799/GTIN: 06260155960213) and omega-3 (IRC: 1228058530/GTIN: 06260155920675) supplements and placebos were procured from Zahravi Pharmaceutical Company (Tabriz, Iran). The supplements and placebos were identical in terms of form, size, color, packaging, smell and taste. In addition, the packages were identified with the letters A, B, C and D and the researcher, participants and statistician were blinded to their content. The participants were assigned to one of the four groups for 8 weeks using block randomization.

Group 1: Omega-3 placebo twice daily and vitamin D placebo every 2 weeks.

Group 2: 1000 mg of omega-3 twice daily and vitamin D placebo every 2 weeks.

Group 3: Omega-3 placebo twice daily and 50,000 IU of vitamin D every 2 weeks.

Group 4: 1000 mg of omega-3 twice daily and 50,000 IU of vitamin D every 2 weeks.

Previous studies had reported no side effects for the use of these doses of the supplements [24, 25].

The participants were contacted on the phone once a week to remind them about taking their medications, to assess their side effects and to ensure the lack of change in their physical activity and food regimen. A table of daily supplement intake was provided to the participants. This table and the empty capsule packs were collected from the women at the end. Moreover, the researcher’s phone number was given to the subjects to call in case of any problems. The subjects were asked not to change their food regimen and physical activity over the eight weeks of the study.

## Measurements

### Questionnaires

Physical activity was assessed using the IPAQ-SF, in which walking and moderate to severe physical activity is expressed as MET-min/week. The level of physical activity was categorized according to the IPAQ criteria as low, moderate, and high [31].

The energy level and nutrients used were measured using the three-day food record (two weekdays and one weekend day) and analyzed in Modified Nutritionist-4 software program (First Databank, San Bruno, CA).

### Anthropometric Indices

Anthropometric indices, including height, weight and waist circumference were measured at the beginning and end of the study. The fasting body weight was measured with no shoes and minimum clothing with accuracy of 0.1 kg using a digital scale (Beurer, BF220-Germany). Height was measured in the standing position, with no shoes and the heels touching the wall, using a stadiometer (Seca, Hamburg, Germany) with accuracy of 0.1 cm. The BMI was determined by dividing weight in kg by height in meters squared. The waist circumference was measured with minimum clothing using an inelastic tape measure at midline between the lowest rib and iliac crest [32].

### Blood samples and biochemical assessments

At the beginning and end of the intervention, 10 cc of venous blood was taken from all the participants after 12 h of fasting. The serum was separated from the clot by centrifuging at 3000 rpm for 10 min and was kept at − 80 °C until the tests were performed [32]. Fasting blood glucose (FBS), cholesterol, triglyceride and HDL-C tests were carried out by spectrophotometry using Pars Azmun (Iran) kit in an auto-analyzer device (BT3000, Italy). The inter-assay and intra-assay CVs of the serum glucose and lipids tests were less than 7%.

LDL-C was determined using Friedewald’s equation [33].

Fasting blood insulin (FBI) and vitamin D were measured by the ELISA technique in Stat Fax 4200 (USA) and a monobind kit (CA.USA). The inter-assay and intra-assay CVs of the vitamin D test were 5% and 6%, respectively, and the inter-assay and intra-assay CVs of the insulin test were 3.5% and 4%, respectively.

The insulin resistance was determined using the HOMA-IR index and the beta-cell function using the HOMA-B index as follows [34]:

$${\text{HOMA}} - {\text{IR}}\, = \,\left[ {{\text{FBI }}\left( {\mu {\text{U}}/{\text{mL}}} \right)\, \times \,{\text{FBS }}\left( {{\text{mg}}/{\text{mL}}} \right)} \right]/ 40 5$$

$${\text{HOMA}} - {\text{B}}\, = \,\left[ {\left( {{\text{FBI }}\left( {\mu {\text{U}}/{\text{mL}}} \right)\, \times \, 3 60} \right)/\left( {{\text{FBS }}\left( {{\text{mg}}/{\text{mL}}} \right) - 6 3} \right)} \right] \% .$$

### Sample size calculation

In a similar study [24], after the intervention, the mean and standard deviation of fasting glucose were 94.6 and 10.30 in the placebo group and 86.8 and 6.40 in the omega-3 and vitamin D co-supplementation group. Based on the relevant formula and taking into account a type I error of 5%, a test power of 80% and an attrition 10%, the sample size was calculated as 42 per group. Finally, 168 subjects entered the study and were divided into four groups.

### Statistical analysis

The present report is based on the intention-to-treat analysis. Normal distribution of continuous variables was assessed by the Kolmogorov–Smirnov test. Continuous and categorical variables were expressed as mean (standard deviation) and number (percentage) respectively. Continuous and categorical variables between groups at baseline were compared using one-way ANOVA and Chi square test respectively.

The two-way mixed ANOVA was used with Bonferroni correction to assess effect of interventions on all outcomes.

Data were analyzed in SPSS-24 software. A P value < 0.05 was considered as statistically significant. All statistical analysis was performed using the intention-to-treat method.

## Results

### Subject characteristics

**Fig. 1:**
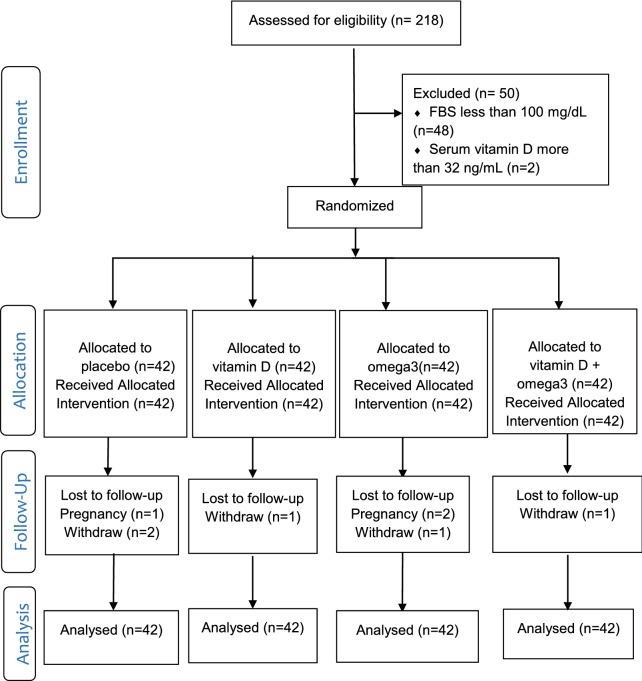
Flow diagram of the study

As shown in Fig. 1, of the 168 participants, three in the placebo group, three in the vitamin D group, one in the omega-3 group and one in the co-supplementation group were excluded from the study. Exclusion was due to pregnancy in three cases and personal reason in five cases. The analysis was performed by the intention-to-treat and per protocol methods; the results obtained were similar. The present report is based on the intention-to-treat analysis of 168 participants. The mean age of the participants was 40.14 ± 7.06 years; 55.4% of the participants had diabetes in first-degree relatives and 53.6% had second-degree relatives with diabetes.

At the beginning of the intervention, there was no significant statistical difference between the four groups in terms of mean age, anthropometric indices, physical activity, food intake, glycemic indices, and serum lipids (Table 1). Physical activity, dietary intake (micronutrients and macronutrients) and time spent out door showed no significant statistical difference within the groups before and after the intervention.

**Table 1:** Baseline characteristics of the participants by group

Characteristic	Placebo group (n = 42)	Omega 3 group (n = 42)	Vitamin D group (n = 42)	Vitamin D + Omega 3 group (n = 42)	P value^¶^
Age (years)	41.85 (7.48)	39.78 (6.88)	39.92 (6.04)	39.00 (7.68)	0.29
Vitamin D	25.47 (5.83)	23.56 (6.42)	21.43(8)	22.03 (6.92)	.44
FBS (mg/dL)	104.52 (6.39)	103.87 (5.88)	103.74 (5.11)	103.71 (4.73)	.98
Insulin (µIU/mL)	7.36 (4.12)	9.14 (3.14)	7.73 (3.66)	10.35 (3.13)	.30
Triglycerides (mg/dL)	123.69 (48.17)	132.10 (66.77)	126.68 (77.7)	137.42 (67.02)	.77
Total cholesterol (mg/dL)	188.59 (32.82)	194.05 (32.4)	197.73 (37.8)	199.35 (42.11)	.40
HDL-c (mg/dL)	48.43 (8.76)	51.08 (8.91)	51.73 (11.47)	48.55 (8.92)	.34
LDL-c (mg/dL)	111 (30.74)	115.36 (26.91)	119.15 (32.71)	122.76 (36.47)	.40
Weight (kg)	74.32 (12.65)	69.65 (9.09)	72.80 (9.23)	72.54 (10.62)	.24
BMI (kg/m^2^)	27.28 (2.74)	27.17 (2.74)	27.01 (2.91)	27.04 (2.86)	.82
Waist circumference (cm)	85.05 (10.42)	82.78 (6.41)	84.6 (8.49)	85.29 (9.85)	.62
Dietary energy intake (kcal)	1639.0 (601.9)	1645.5 (451.8)	1523 (499.6)	1570.60 (509.8)	.26
Physical activity (MET-min/week)	698.19 (618.83)	815.34 (778.54)	706.78 (656.76)	808.66 (766.99)	.85
Time spent out door (h/day)	1.7 (1.8)	1.8 (1.2)	1.8 (1.4)	1.8 (1.8)	.88

BMI: Body Mass Index; FBS: Fasting blood sugar; HDL-C: High-density lipoprotein cholesterol; HOMA-IR: Homeostasis Model Assessment estimated Insulin Resistance; HOMA-B: homeostatic model assessment for B-cell function-B; LDL-C: Low-density lipoprotein cholesterol

All values are means (SDs)

*P value is significant

^¶^ Obtain from One-way analysis of variance

No side effects were reported following the consumption of supplements or placebos.

### Effects on glycemic indices

Tables 2 and 4 present the effects of supplements intake on glycemic status.

**Table 2:** Comparison of glycemic indices and Vitamin D in each group before and after the intervention

Variables	Time	Group 1 Placebo group (n = 42)	Group 2 Omega 3 group (n = 42)	Group 3 Vitamin D group (n = 42)	Group 4 Vitamin + Omega 3 group (n = 42)
Vitamin D	Before	25.47 (5.83)	23.56 (6.42)	21.43 (8)	22.03 (6.92)
	After	20.97 (6.63)	21.17 (7.40)	29.71 (12.11)	31.71 (11.51)
	P-value^¶^	< .001*	< .001*	< .001*	< .001*
FBS (mg/dL)	Before	104.52 (6.39)	103.87 (5.88)	103.74 (5.11)	103.71 (4.73)
	After	107.12 (9.94)	97.23 (5.87)	100.18 (8.94)	94.33 (8.02)
	P-value^¶^	< .001*	< .001*	< .001*	< .001*
Insulin (µIU/mL)	Before	7.36 (4.12)	9.14 (3.14)	7.73 (3.66)	10.35 (3.13)
	After	10.42 (4.89)	7.34 (3.53)	9.65 (4.08)	7.73 (2.4)
	P-value^¶^	< .001*	< .001*	< .001*	< .001*
HOMA-IR	Before	1.91 (1.13)	2.34 (0.82)	1.98 (0.94)	2.65 (0.83)
	After	2.75 (1.34)	1.75 (0.78)	2.38 (1.07)	1.79 (0.6)
	P-value^¶^	< .001*	< .001*	< .001*	< .001*
HOMA-B	Before	0.64 (0.34)	0.82 (0.56)	0.69 (0.33)	0.95 (0.42)
	After	0.92 (0.64)	0.81 (0.29)	0.96 (0.4)	0.92 (0.28)
	P-value^¶^	< .001*	.92	< .001*	0.04*

FBS: Fasting blood sugar; HOMA-IR: Homeostasis Model Assessment estimated Insulin Resistance; HOMA-B: homeostatic model assessment for B-cell function-B

All values are means (SDs)

*P values is significant

^¶^ Obtain from paired samples t-test

A significant group*time interaction was observed for fasting insulin and glucose, HOMA-IR and HOMA-B changes (P < 0.001).

The fasting glucose levels decreased significantly in the groups 2, 3 and 4 at the end of the study. A significant statistical difference was observed in fasting glucose changes between the co-supplementation group and the other three groups (P < 0.05).

At the end of the intervention the fasting insulin levels decreased significantly in the co-supplementation group and the omega-3 group (P < 0.001), but increased in the placebo and vitamin D groups. A significant statistical difference was observed between the co-supplementation group and the other three groups in terms of fasting insulin changes (P < 0.05).

The co-supplementation group and the omega-3 group showed a significant reduction in HOMA-IR at the end of the study (P < 0.001). There was no other statistically significant differences between groups in HOMA-IR.

At the end of the intervention, the decrease in HOMA-B was observed in the co-supplementation and omega-3 groups. There was also a significant statistical difference (P < 0.05) between the co-supplementation group and the other three groups in HOMA-B changes (P < 0.05).

### Effects on lipid profiles

Tables 3 and 4 show the effects of supplements intake on serum lipids.

**Table 3:** Comparison of anthropometric indices and lipid profiles in each group before and after the intervention

Variables	Time	Group 1 Placebo group (n = 42)	Group 2 Omega 3 group (n = 42)	Group 3 Vitamin D group (n = 42)	Group 4 Vitamin + Omega 3 group (n = 42)
Triglycerides (mg/dL)	Before	123.69 (48.17)	132.10 (66.77)	126.68 (77.7)	137.42 (67.02)
	After	134.51 (63.45)	116.62 (60.02)	129.95 (74.65)	115.09 (53.36)
	P-value^¶^	< .001*	< .001*	0.34	< .001*
Total cholesterol (mg/dL)	Before	188.59 (32.82)	194.05 (32.4)	197.73 (37.8)	199.35 (42.11)
	After	184.37 (34.09)	179.75 (30.76)	187.49 (32.61)	176.61 (40.16)
	P-value^¶^	.23	< .001*	< .001*	< .001*
HDL-c (mg/dL)	Before	48.43 (8.76)	51.08 (8.91)	51.73 (11.47)	48.55 (8.92)
	After	48.10 (9.32)	51.88 (51.88)	52.23 (10.55)	49.76 (10.17)
	P-value^¶^	.12	.01*	.11	< .001*
LDL-c (mg/dL)	Before	111 (30.74)	115.36 (26.91)	119.15 (32.71)	122.76 (36.47)
	After	106.88 (29.81)	103.82 (28.98)	108.85 (28.04)	103.71 (35.52)
	P-value^¶^	.26	< .001*	< .001*	< .001*
Weight (kg)	Before	74.32 (12.65)	69.65 (9.09)	72.80 (9.23)	72.54 (10.62)
	After	74.64 (12.81)	69.58 (9.23)	72.86 (9.4)	72.17 (10.41)
	P-value^¶^	< .001*	.45	.38	< .001*
BMI (kg/m^2^)	Before	27.28 (2.74)	27.17 (2.74)	27.01 (2.91)	27.04 (2.86)
	After	27.27 (2.96)	27.09 (2.77)	26.98 (2.91)	26.99 (2.91)
	P-value^¶^	.94	.13	.13	< .001*
Waist circumference (cm)	Before	85.05 (10.42)	82.78 (6.41)	84.60 (8.49)	85.29 (9.85)
	After	85.04 (11.05)	81.94 (6.73)	84.17 (8.69)	84.13 (9.16)
	P-value^¶^	.88	< .001*	< .001*	< .001*

BMI: Body Mass Index; HDL-C: High-density lipoprotein cholesterol; LDL-C: Low-density lipoprotein cholesterol

All values are means (SDs)

*P value is significant

^¶^Obtain from paired samples t-test

**Table 4:** Adjusted changes in glycemic indices, lipid profiles and anthropometric indices in each group

Variables	Group 1 Placebo group (n = 42)	Group 2 Omega 3 group (n = 42)	Group 3 Vitamin D group (n = 42)	Group 4 Vitamin + Omega 3 group (n = 42)	P value^¥^		ηp2^¥^	
					Time	Time* group interaction	time	Time* group interaction
Vitamin D	− 4.49 (5.03)	− 2.38 (5.28)	8.27 (7.77)	9.68 (7.34)^#^	< .001*	< .001*	.15	.49
FBS (mg/dL)	2.60 (6.59)	− 6.64 (5.14)	− 3.56 (7.11)	− 9.37 (6.4)^ǁ^	< .001*	< .001*	.3	.33
Insulin (µIU/mL)	3.06 (3.69)	− 1.80 (2.16)	1.92 (3.91)	− 2.61 (1.68)^ǁ^	.13	< .001*	.002	.38
HOMA-IR	0.83 (1.05)	− 0.58 (0.51)	0.40 (1.05)	− 0.85 (0.48)	.04*	< .001*	.004	.42
HOMA-B	0.28 (0.53)	− 0.002 (0.42)	0.27 (0.4)	− 0.04 (0.31)^ǁ^	< .001*	< .001*	.08	.19
Triglycerides (mg/dL)	10.81 (54.27)	− 15.48 (40.02)	3.27 (56.55)	− 22.32 (34.58)	< .001*	< .001*	.01	.07
Total-cholesterol (mg/dL)	− 4.21 (22.71)	− 14.29 (26.8)	− 10.23 (29.89)	− 22.74 (29.7)	< .001*	< .001*	.18	.006
HDL-C (mg/dL)	− 0.32 (3.74)	0.82 (5.35)	0.4 (5.15)	1.29 (5.68)^ǁ^	< .001*	.004*	.01	.01
LDL-C (mg/dL)	− 4.12 (20.58)	− 11.53 (25.52)	− 10.29 (26.40)	− 19.04 (29.03)	< .001*	< .001*	.16	.04
Weight	0.31 (1.03)	− 0.06 (1.42)	0.06 (1.27)	− 0.36 (1.65)^ǁ^	.77	< .001*	0	.03
BMI	− 0.002 (0.54)	− 0.07 (0.81)	− 0.036 (0.63)	− 0.05 (0.68)	.04	.63	.004	.002
Waist circumference	− 0.01 (1.7)	− 0.84 (1.77)	− 0.43 (1.8)	− 1.15 (2.23)^ǁ^	< .001*	< .001*	.09	.05

All values are means (SDs)

*P value is significant

^¥^ Obtain from two way repeated measures ANOVA after adjusting for covariates

^#^ Significant difference with group 1 and 2

^ǁ^ Significant difference with and other 3 groups

(¥, ǁ, #) Obtain from Two way repeated measures ANOVA with Bonferroni correction

ηp2 (partial eta squared) = 0.14 or more are large effects, 0.06 to 0.14 are medium effects and Less than 0.6 are small effects

A significant group*time interaction was observed for the changes in cholesterol, triglyceride, HDL-C and LDL-C (P < 0.05).

Although, at the end of the study, there was a significant reduction in the groups 2, 3 and 4 in terms of total cholesterol (P < 0.05), there were no significant differences in the changes in total cholesterol between the groups.

There was a significant reduction in triglyceride (P < 0.001) after the intervention in the co-supplementation group and the omega-3 group, but no other statistically significant difference was observed in triglyceride changes between the groups.

At the end of the study, the HDL-C level had increased significantly in the groups 2, 3 and 4 (P < 0.05). There was a statistically significant difference between the co-supplementation group and the other three groups in HDL-C changes (P < 0.05).

Despite the fact that LDL-C levels decreased significantly in the groups 2, 3 and 4 after the intervention (P < 0.001), there was no statistically significant difference between the groups in LDL-C changes.

### Effects on anthropometric indices

The effects of supplements intake on anthropometric indices is shown in Tables 3 and 4. A significant group*time interaction was observed for waist circumference and weight changes (P < 0.001). After the intervention, statistical significant decrease in weight was observed in the co-supplementation group. Weight decreased in the omega-3 group and increased in the other two groups; however, the changes were not statistically significant. There was a significant difference in terms of weight changes between the co-supplementation group and other three groups (P < 0.05). Although BMI had more decreased in the co-supplementation group and the omega-3 group, no statistically significant difference was observed in BMI changes between the groups.

At the end of the study, waist circumference had decreased significantly in the groups 2, 3 and 4 (P < 0.001). There was a significant difference between the co-supplementation group and other three groups in waist circumference changes (P < 0.05).

After adjusting for covariates (age, BMI, physical activity level and food intake), no change was observed in results of the repeated measures ANOVA for any of these outcomes.

## Discussion

The present study demonstrated that vitamin D and omega-3 co-supplementation for 8 weeks in women of reproductive age with pre-diabetes and hypovitaminosis have positive effects on fasting blood insulin, glucose and HDL-C levels, HOMA-B, waist circumference and weight. Research has shown that an increased fasting glucose in pre-diabetic individuals can lead to diabetes type 2 in the long term [35]. Therefore, Vitamin D and omega-3 co-supplementation can be considered for preventing diabetes type 2 because of their positive effects on fasting blood glucose and insulin.

Various studies have shown that vitamin D has a role in the metabolism of carbohydrates [36] and fats [37–39]. Several mechanisms have been proposed for this process. Vitamin D affects Parathyroid Hormone (PTH) secretion and its deficiency results in an increase in PTH. A rise in this hormone is associated with an increase in insulin resistance and a reduction in lipolysis [38].

This vitamin can activate the Insulin Receptor (INSR) synthesis gene by binding to the nuclear receptor, thus enhancing the synthesis of these receptors and increasing the presence of insulin-regulated glucose transporter type-4 (GLUT4) in the cell membrane [40]. Vitamin D increases the expression of the peroxisome proliferator-activated receptor gamma (PPAR-γ) gene, which improves fatty acid metabolism and increases insulin sensitivity. These vitamin receptors are present in the pancreatic beta cells and the immune system. Therefore, vitamin D deficiency affects insulin secretion, peripheral responses to insulin, and immune system function, which can result in insulin resistance and other inflammatory processes contributing to glucose and fat metabolism disorders [40]. Vitamin D modulates the function of the renin-angiotensin system by decreasing the renin gene expression and inhibiting angiotensin-1 receptors. The increased activity of this system causes insulin resistance, inflammation and hypertension [40].

A number of studies have suggested that high doses of vitamin D do not have beneficial effects on glycemic indices and other cardiovascular risk factors in pre-diabetic individuals [35, 41–43].

The findings of present study in the vitamin D group were similar to the results reported by Solids [35]. Although a significant reduction was found in fasting blood glucose, total cholesterol and LDL-C levels, no statistically significant differences were observed in other lipids and insulin metabolism markers. Also, in a meta-analysis of ten clinical trials in persons with pre-diabetic, Poolsup et al. showed that vitamin D intake reduced fasting glucose significantly but did not affect insulin resistance [44].

Moreover, some studies have shown that unsaturated fatty acids are effective in a number of chronic diseases including cardiovascular diseases, type 2 diabetes, and the metabolic syndrome [45, 46].

Animal model studies reported the mechanism of effect of omega-3 on glycemic indices and serum lipids in three main areas: (1) Some adipokines such as adiponectin and leptin, stimulate AMP-activated protein kinase (AMPK) in the tissues. AMPK reduces lipogenesis and increases the oxidation of fatty acids. It stimulates glucose transport into the cell independent of insulin. Omega-3 can activate AMPK in white adipose tissues, muscles and the liver. (2) Omega-3 reduces glucose synthesis and lipogenesis in the liver. (3) Omega-3 increases anti-inflammatory synthesis, such as resolvin E1, resolvin D1 and protectin D1, thus reducing oxidative stress and the cell damage caused by them. Omega-3 with anti-inflammatory effects, increased insulin sensitivity and reduced adipocyte proliferation and hence reduces the body lipid content, thus resulting in decreased insulin and serum lipids [47].

Different results have been reported from clinical trials on omega-3 in pre-diabetic individuals. Some studies have reported beneficial effects for omega-3 in improving fasting glucose, insulin resistance, triglyceride levels, and waist circumference [48, 49]. A number of studies have found that omega-3 has little or no effect on fat and glucose metabolism but has significant effects on triglyceride levels [47, 50].

In the present study, at the end of the intervention, the omega 3 group showed a significant improvement in glycemic indices and serum lipids compared to the beginning of the study. This disparity in results may be due to the differences in sample size [50], gender and age of the participants [48, 49] and doses administered [48, 50]. Furthermore, a study have shown that omega-3 can activate vitamin D [51]. Therefore, various studies were carried out on effectiveness of vitamin D and omega-3 co-supplementation on glycemic indices.

Jamilian et al. investigated the effects of the intake of 50,000 IU of vitamin D every 2 weeks and 1000 mg of omega-3 twice daily in women with gestational diabetes for 6 weeks. This co-supplementation was reported to have positive effects on glycemic control, triglyceride and VLDL-c level, but had no effects on other serum lipids [24]. These results were consistent with the present findings in terms of the effects of vitamin D and omega-3 co-supplementation on fasting insulin and glucose, but inconsistent in terms of serum lipids, perhaps due to the differences in study participants. The sample studied by Jamilian included pregnant women with gestational diabetes while the sample in the present study consisted of women of reproductive age with pre-diabetes. Hormonal changes during pregnancy cause hyperlipidemia and increase insulin resistance, resulting the lipids to be used as an energy source in pregnant women, which it does not exist healthy non-pregnant women [7]. Therefore, vitamin D and omega-3 co-supplementation had more significant effect on glycemic status and serum lipids in pregnant women with gestational diabetes.

In line with the present findings, Gurol et al. also observed the synergistic effects of vitamin D and omega-3 co-supplementation in rats undergoing islet transplantation, the glycemic level decreased in the co-supplementation group [52].

Baidal et al. investigated the effects of high doses of omega-3 (two 1-g capsules of omega-3, three times daily) and high doses of vitamin D (25,000 IU of vitamin D weekly) in a 14-year-old adolescent with diabetes type 1 [25]. Fasting glucose was found to be lower in the first-year follow-up compared to the baseline, and the researchers suggested that this co-supplementation could be effective in beta cell function. The results were similar to findings of the present study.

Davis et al. also studied the effects of the daily intake of four to ten grams of fish oil and 2000 IU of vitamin D daily on serum lipids in 45 men and women with subclinical arteriosclerosis over 18 months [53]. They reported a reduction in cholesterol, triglyceride and LDL-C and an increase in HDL-C in the subjects. The difference between the results of this study and the present study might be due to the higher dose of omega-3 administered and the longer intervention period.

Effectiveness of this co-supplementation on glycemic control and lipid profiles was previously shown in patients with gestational diabetes, diabetes type 1, and atherosclerosis [24, 25, 53]. To the best of our knowledge, the present study is the first on the effect of vitamin D and omega-3 co-supplementation on glycemic index and lipid profiles in pre-diabetic women. The results suggest that this co-supplementation can improve fasting serum glucose, insulin and HDL-C and beta cell function in reproductive-aged women with pre-diabetes and hypovitaminosis D.

### Strengths and limitations

This study had several strengths. (1) It was a randomized, triple-blind, factorial design, clinical trial with an appropriate sample size for determining a reasonable effect size.

(2) The two-way repeated-measures ANOVA and Bonferroni correction were used to determine the interaction of co-supplementation on glycemic status and lipid profiles changes and to compare the groups. (3) To control physical activity and food intake, two questionnaires were administered at the beginning and end of the intervention, and weekly phone conversations were held with participants to remind them about these two issues and to assess their status of supplement intake and side effects. One of the limitations of this study was the short duration of the intervention, as this period could not necessarily have reflected the long-term effects of using these supplements; therefore, the effectiveness of this co-supplementation in delaying diabetes was not investigated.

## Conclusion

Vitamin D and omega 3 co-supplementation improves fasting blood glucose, insulin and HDL-C levels and HOMA-B in reproductive-aged women with pre-diabetes and hypovitaminosis D and therefore can be recommended for glycemic control in these individuals.

## Data Availability

The datasets used during the current study are available from the corresponding author on reasonable request.

## Abbreviations

BMI: Body mass index
DM: Diabetes mellitus
FBS: Fasting blood sugar
FBI: Fasting blood insulin
HDL-C: High-density lipoprotein cholesterol
HOMA-IR: Homeostasis model assessment estimated insulin resistance
HOMA-B: Homeostatic model assessment for B cell function-B
IPAQ: International Physical Activity Questionnaire
LDL-C: Low-density lipoprotein cholesterol
T2DM: Type 2 Diabetes Mellitus
TC: Total Cholesterol
TG: Triglyceride
